# Excessive Food Intake, Obesity and Inflammation Process in Zucker *fa/fa* Rat Pancreatic Islets

**DOI:** 10.1371/journal.pone.0022954

**Published:** 2011-08-03

**Authors:** Myriam Chentouf, Gregor Dubois, Céline Jahannaut, Françoise Castex, Anne Dominique Lajoix, René Gross, Sylvie Peraldi-Roux

**Affiliations:** 1 Centre National de la Recherche Scientifique – Formation de Recherche en Evolution 3400, Centre de Pharmacologie et Innovation pour le Diabète, Faculté de Pharmacie, Montpellier, France; 2 Institut de Recherche pour le Développement, Unité Mixte de Recherche, Faculté de Pharmacie, Montpellier, France; Universita Magna-Graecia di Catanzaro, Italy

## Abstract

Inappropriate food intake-related obesity and more importantly, visceral adiposity, are major risk factors for the onset of type 2 diabetes. Evidence is emerging that nutriment-induced β-cell dysfunction could be related to indirect induction of a state of low grade inflammation. Our aim was to study whether hyperphagia associated obesity could promote an inflammatory response in pancreatic islets leading to ß-cell dysfunction. In the hyperphagic obese insulin resistant male Zucker rat, we measured the level of circulating pro-inflammatory cytokines and estimated their production as well as the expression of their receptors in pancreatic tissue and β-cells. Our main findings concern intra-islet pro-inflammatory cytokines from *fa/fa* rats: IL-1β, IL-6 and TNFα expressions were increased; IL-1R1 was also over-expressed with a cellular redistribution also observed for IL-6R. To get insight into the mechanisms involved in phenotypic alterations, abArrays were used to determine the expression profile of proteins implicated in different membrane receptors signaling, apoptosis and cell cycle pathways. Despite JNK overexpression, cell viability was unaffected probably because of decreases in cleaved caspase3 as well as in SMAC/DIABLO and APP, involved in the induction and amplification of apoptosis. Concerning β-cell proliferation, decreases in important cell cycle regulators (Cyclin D1, p35) and increased expression of SMAD4 probably contribute to counteract and restrain hyperplasia in *fa/fa* rat islets. Finally and probably as a result of IL-1β and IL-1R1 increased expressions with sub-cellular redistribution of the receptor, islets from *fa/fa* rats were found more sensitive to both stimulating and inhibitory concentrations of the cytokine; this confers some physiopathological relevance to a possible autocrine regulation of β-cell function by IL-1β. These results support the hypothesis that pancreatic islets from prediabetic *fa/fa* rats undergo an inflammatory process. That the latter could contribute to β-cell hyperactivity/proliferation and possibly lead to progressive β-cell failure in these animals, deserves further investigations.

## Introduction

The prevalence and incidence of type 2 diabetes (T2D) are dramatically increasing worldwide in both developed and developing countries. This multifactorial disease results from the interaction of environmental factors and genetic predisposition leading to two major abnormalities: insulin resistance and defective β-cell function. During the long lasting silent phase, known as prediabetes, that precedes the onset of T2D, hyperinsulinemia compensates for insulin resistance. Hyperglycemia then develops with a progressive β-cell dysfunction, but the mechanisms involved remain to be determined.

In this context, inappropriate food intake and related obesity are major risk factors for the onset of T2D. High carbohydrate and high fat diets, the major cause of obesity, represent two diabetogenic factors that can lead, by their own, to β-cell dysfunction.

The molecular mechanisms that link obesity and insulin resistance to ß-cell dysfunction have not been completely understood yet and are the subject of intensive research. Growing evidence suggests that obesity, insulin resistance and T2D are accompanied by a state of subclinical inflammation [1], [2]. Indeed, biomarkers of inflammation such as leucocyte count, tumor necrosis factor α (TNFα), interleukin-6 (IL-6) and C-reactive protein are increased in obesity and predict the development of T2D [3], [4], [5], [6].

In addition, cytokines which are crucially involved in the etiopathology of type 1 diabetes [7], [8], also play a role in islet dysfunction in T2D. In rodents, high-fat feeding leads to increased adipocyte expression of monocyte chemotactic protein-1 (MCP-1) which could contribute to the stimulation of macrophage infiltration into adipose tissue [9], [10]. Evidence also accumulates that changes in cytokine production by the liver, adipose tissue and infiltrating cells in response to chronic exposure to lipids and glucose play an important role as pathogenic factors in the development of T2D. Concerning pancreatic ß-cell, high glucose and IL-1ß autostimulation have been shown to increase IL-1ß mRNA and protein expression in human islets. Furthermore characterization of an increased IL-1ß expression in pancreatic sections of patients with T2D and hyperglycaemic Psammomys obesus gerbils, have led to the hypothesis that intra-islet expression of inflammatory cytokines and especially IL1ß, contribute to the pathogenesis of T2D [11], [12], [13].

Even if data from animal models of T2D support the concept that local inflammation processes are essential promoters in the disease pathogenesis, further studies are required to better characterize intra-islet inflammation and to determine whether overfeeding and related obesity could exacerbate and prompt ß-cell to express cytokines and their receptors contributing thereby to defects in insulin secretion and ß-cell survival.

In this context and using the hyperphagic obese Zucker *fa/fa* rat as a relevant model, our aim was to evaluate to what extent the low grade inflammation state induced by excessive caloric intake could lead to ß-cell dysfunction in the early phase of T2D. We studied the effect of excessive food intake, first, on the plasma levels of circulating pro-inflammatory cytokines, and second, on the ß-cellular expression of cytokines, of their receptors and signalling pathways factors. Furthermore, to mimic and appreciate the impact of possible autocrine effects of IL-1ß on ß-cell function and survival, we investigated and compared the effects of the cytokine on insulin release and apoptosis in *fa/+* and *fa/fa* Zucker rat islets.

## Materials and Methods

### Materials

In the immunofluorescence studies we used anti-cytokines antibodies against IL-1ß, IL-6, TNFα, IFNγ, and anti-cytokine receptor antibodies against IL-1R1, IL-1R2, IL-6R, TNF-R1, IFN-Rα, IFN-Rß (Santa Cruz Biotechnology, Santa Cruz, CA); guinea pig anti-insulin antibody was from MP Biomedicals (MP Biomedicals, Irvine, CA). Fluorescein isothiocyanate (FITC)-conjugated anti–guinea pig, Texas Red-conjugated anti-rabbit and anti-goat (Vector Laboratories, Burlingame, CA), were used as secondary antibodies. Western blot experiments were performed with mouse anti-phospho-ERK1/2 (pT202/pY204), anti-phospho-JNK/SAPK (pT183/pY185), anti-IKKγ, rabbit anti-Bcl-x (BD Biosciences), rabbit anti-ERK, anti-JNK, anti-Cleaved caspase-3, anti-Bcl-10 (Cell Signaling), rabbit anti-Caspase-1- and mouse anti-GRB2- (Abcam Cambridge, UK) antibodies. For apoptosis detection, rabbit polyclonal anti-annexin V antibody (abcam) was used at 5 µg/ml. Recombinant rat IL-1ß was purchased from R & D Systems (Minneapolis, MN).

### Animals

6 week-old Male Zucker *fa*/*fa* obese and Zucker *fa/+* lean rats were purchased from Harlan. They had free access to a standard laboratory chow diet and water during 2 to 3 weeks. Body weight was measured weekly. Blood was collected from the tail vein, centrifuged and plasma aliquots sampled and frozen, once a week and on the day of the experiments; animals were then sacrificed by decapitation and tissues were immediately isolated.

Animals were treated according to institutional guideless for animal use and care. The animals have been handled in the laboratory animal house and used in accordance with the “Principles of Laboratory Animal Care” (NIH Publication no. 85-23, revised 1985) and according to national law. Our laboratory is habilitated to perform experimentations on alive vertebrate animals (approval C34-172-25 from the French Agriculture Ministry). Our study is approved by the ethics committee of our institution.

### Cytokine measurement

Circulating cytokines have been identified using the Chemiarray rat cytokine kit from Chemicon (Canada), allowing ?identification of GM-CSF, IFNα, IL-1β, IL-4, IL-6, IL-10, LIX, Leptin, MCP-1, MIP-3α, β-NGF, TIMP-1, TNF-α, VEGF, Fractalkin, CNTF, CINC-3, and CINC-2. Two fold diluted plasma samples (4 *fa/+*, and 4 *fa/fa* Zucker rats) were incubated with saturated antibody membranes at room temperature for 2 hours. After washing, biotin-conjugated anti-cytokine primary antibody was added to the membranes and further incubated during 2 hours at 4°C with HRP-conjugated Streptavidin. Membranes were then exposed to X-ray film and the signal analyzed. Relative levels of cytokines were evaluated by comparing the signal intensities between *fa/fa* and *fa/+* Zucker rats groups. The intensities of signals were quantified directly with a chemiluminescence imager (Vilbert-Lourma).

Rat serum samples were simultaneously tested for cytokines IL-1α, IL-1β, IL-2, IL-4, IL-6, IL-10, GM-CSF, interferon-γ, and TNF-α using a rat cytokine 9-Plex assay (Bio-Plex; Bio-Rad, Hercules, CA). The assay was run according to the manufacturer's instructions. In brief, the premixed standards were reconstituted in 0.5 ml of a Bio-Plex human serum standard diluent, generating a stock concentration of 50,000 pg/ml for each cytokine. The standard stock was serially diluted in the Bio-Plex rat serum standard diluent to generate eight points for the standard curve. The assay was performed in a 96-well filtration plate supplied with the assay kit. Premixed beads (50 µl) coated with target capture antibodies were transferred to each well of the filtration plate and washed twice with Bio-Plex wash buffer. The samples were diluted 1∶3 in the Bio-Plex serum sample diluent. Premixed standards or diluted samples (50 µl) were added to each well containing washed beads. The plate was shaken and incubated at room temperature for 30 min at low speed (300 rpm). After incubation and washing, premixed biotin conjugated detection antibodies were added to each well. Then, the plate was incubated for 30 min with shaking at low speed (300 rpm). After incubation and washing, streptavidin-phycoerythrin was added to each well. The incubation was terminated after shaking for 10 min at room temperature. After washing, the beads were resuspended in 125 µl of Bio-Plex assay buffer. Beads were read on the Bio-Plex suspension array system (Bio-Rad), and the data were analyzed using Bio-Plex Manager software version 3.0 with 5PL curve fitting.

### Isolation of rat pancreatic islets

Male Zucker *fa/*+ (260–280 g) and *fa/fa* (350–380 g) rats were used in all experiments. After collagenase (Roche, Meylan, France) perfusion, pancreas was removed and islets of Langerhans were isolated using Ficoll gradients (Sigma Aldrich) as previously described [14]. Rat islets were hand-picked and then used for mRNA and protein extraction, as well as islets functional and ß-cell immunofluorescence studies.

### RNA extraction, reverse transcription and real time PCR

Total RNA from isolated rat islets was extracted with the Trizol reagent (Invitrogen, Carlsbad, CA) and purified using the purification Qiagen kit (Qiagen, Germany). First-strand cDNA was obtained from 5 µg total RNA using 3 µg random hexanucleotide primers (Invitrogen), 1 µg oligo(dT) (Invitrogen), and Superscript II RNAse H^−^ Reverse Transcriptase (Invitrogen) in a volume of 50 µl. Quantitative detection of PCR products was performed using a 10× LightCycler Faststart DNA Master Sybr Green I mix (Roche), 5 µM of each primer, and 2.5 mmol/l MgCl_2_. After an 8-min denaturation at 95°C, 50 cycles were performed: 95°C for 5 s, 64°C for 10 s, and 72°C for 6 s. As control, a melting curve was performed after each amplification. A standard curve was made with serial dilutions (H_2_O; 1∶6, 1∶36, 1∶216, 1∶1,296, and 1∶7,776) of a control cDNA sample to evaluate the efficiency of the primers and to relatively quantify the expression level of each sample. The relative expression was normalized using the ß actin gene as housekeeping gene. Oligonucleotide primers were as follows: rat IL-1ß: forward 5′-GGC TTC GAG ATG AAC AAC AAA -3′ and reverse 5′-AGA ATA CCA CTT GTT GGC TTA -3′, rat IL-1R1: forward 5′-AGG GAC AGA CCT GTG ATT A-3′ and reverse 5′-TTC CAG TAG ACA AGG TCG G-3′, rat IL-1R2: forward 5′-TAT GAC ATT TAC CTA CGA GGG-3′ and reverse 5′-AAC GTG CTG TTA GCC A-3′, rat TNFα: forward 5′-GGT GAT TGG TCC CAA CA-3′ and reverse 5′- GTC TTT GAG ATC CAT GCC-3′, rat TNFα-R1: forward 5′- CTT TCT AAG CGG AAA TGA G-3′, and reverse 5′-TCG GCA CAG TAG ACT GA-3′, rat TNFα-R2 forward: 5′-GAT GTT AGG ACT GGC GA-3′ and reverse 5′-CGC TGT GAC TCT TGC T -3′, rat IL6 forward: 5′-AAC AGC GAT GAT GCA C-3′ and reverse 5′-TGG GGT AGG AAG GAC T-3′, rat IL6-R forward: 5′-ATG ACA ACC ACG AGG A-3′ and reverse 5′-GGA AGG TCG GCT TCA G-3′, IFNγ-R1 forward: 5′-ATT TGG ATG CTG CTT GT-3′ and reverse 5′-CAG GTT TGG TCT CGG A-3′.

### Immunofluorescence studies on ß-cells and pancreatic tissue

After islets digestion with trypsine/EDTA, isolated pancreatic ß-cells were grown during 72 hours and seeded on LabTech chamber slide system (Sigma) previously coated with poly-L-lysine at 0.1 mg/ml (Sigma). Pancreatic-cells were cultured in RPMI medium containing 10% of fetal calf serum (FCS), 50 U/mL penicillin, 50 µg/mL streptomycin, 2 mmol/L glutamine (Life technology, France), 1 M hepes, 100 mM sodium pyruvate, 50 mM 2-mercaptoethanol and glucose (5.6 mM and 4,2 mM for respectively *fa/+* and *fa/fa* pancreatic ß-cells).

Then, cells were washed with PBS (pH 7.4) containing CaCl_2_ and MgCl_2_, fixed with 3% paraformaldehyde for 30 min, permeabilized 5 min in 0.1% Triton X-100 and quenched with 50 mM NH_4_Cl for 10 min. After two washings with PBS, the slides were saturated with 2% BSA-Gelatine solution and then incubated with a combination of primary antibodies (anti-cytokine or anti-cytokine receptor antibody diluted at 1/50 plus anti-insulin antibody diluted at 1/200) overnight at 4°C. After three washings, cells were incubated for 1 h with FITC- and Texas red- conjugated antibodies. After three additional washings, cells were covered with citifluor (Citifluor, U.K.) and observed with the BioRad MRC 1024 confocal microscope using the facilities of RIO imagery plateform (Montpellier, France). The negative control was performed by incubating the cells with the secondary antibody alone.

Frozen pancreatic tissue sections (5 µm) from *fa/fa* and *fa/+* were fixed, permeabilized and quenched as previously described. After several washings, the slides were incubated with anti-cytokine or anti-cytokine receptor antibodies. After incubation with conjugated antibodies, slides were observed with an upright fluorescence microscope using the facilities of RIO imagery platform. The negative control was performed using only the conjugated antibody.

### Insulin secretion by isolated islets

Following isolation, 20 islets per batch were incubated overnight in RPMI medium supplemented with FCS and adjuvant (as described) plus 10 mM glucose for *fa/+* islets and 7.5 mM glucose for *fa/fa* islets to compensate for the higher sensitivity and responsiveness of the latter; they were then cultured for 2 days in the absence or in the presence of increasing IL-1β concentrations of (0.01, 0.1, 1 and 10 ng/ml). At the end of the 48 h exposure period, islets were then washed, deprived in glucose during 1 hour and incubated in the presence of 2.8 and 8.3 mM glucose. Islet supernatant fractions were then collected and insulin was extracted from islets with acid/alcohol mixture (1.5%/75%). Quantification of insulin content and insulin present in islet supernatants was performed using HTRF assay (Cisbio). Differences in insulin release between 2.8 and 8.3 mM glucose were estimated for each experimental condition and plotted on the corresponding figure, as percentages of the increase recorded without exposure to the cytokine.

### Apoptosis detection

Annexin V is a Ca^2+^-dependent phospholipid-binding protein with a high affinity for phosphatidylserine (PS). In normal cells, PS is located on the cytoplasmic surface of the cell membrane. In apoptotic cells, PS is translocated from the inner to the outer surface of the cell membrane. Annexin V labeled with a fluorophore can identify apoptotic cells by binding to PS exposed on the outer leaflet. Pancreatic ß-cells cultured in LabTech chamber slide (as previously described) were treated with vehicle or with IL-1-ß (0.01; 0.1; 1; 10 ng/ml) for 48 hours and then washed once with ice-cold PBS, immunostained with rabbit polyclonal anti-annexin V antibody (abcam) and then fixed with 3% paraformaldehyde for 30 min. Cells were washed again and analyzed by confocal microscopy with excitation at 488 nm (green, annexin V).

### Protein extraction and Western-blotting

Isolated islets from *fa/+* or *fa/fa* zucker rats were homogenized in 20 mmol/l Tris lysis buffer, pH 7.4, containing 150 mmol/l NaCl, 1% Triton X-100, 0.5% NP-40, and a cocktail of protease inhibitors (Roche Applied Science, Mannheim, Germany). Insoluble materials were removed by centrifugation. The proteins (40 µg) were fractionated on a 12% polyacrylamide gel and transferred to a nitrocellulose membrane. After blocking with 5% dried skim milk or BSA, filters were then incubated overnight with anti-phospho-ERK1/2, anti-ERK1, anti-phospho-JNK/SAPK, anti- JNK anti-IKKγ (diluted 1/250), anti-Caspase-1, anti-GRB2, anti-cleaved caspase-3, anti Bcl-x and Bcl-10 antibodies (1/1000). After three washings, membranes were incubated with a horseradish peroxidase–conjugated anti-mouse or rabbit antibody (diluted 1∶3,000; Sigma-Aldrich, Steinheim, Germany). Immunoreactivity was detected using an enhanced chemiluminescence reaction (Amersham Biosciences, Little Chalfont, U.K) and analysed with a chemiluminescence imager.

### Antibody Arrays

Panorama antibody microarray containing 224 different antibodies spotted in duplicate on nitrocellulose-coated glass slides was purchased from Sigma-Aldrich. Protein extracts (1 mg/ml) from zucker *fa/+* or *fa/fa* rat islets were labeled with Cy3 and Cy5 (Amersham Biosciences) as described by the manufacturer (Sigma). Samples labeled with a dye/protein molar ratio >2 were applied to the antibody microarray in Array Incubation Buffer (Sigma) and incubated for 45 min protected from light with gentle shaking. The array was then washed three times with 5 ml of Washing Buffer (Sigma) and air-dried. Cy3 and Cy5 signals were read on the Gene Pix Pro 4.0 (MDS Analytical Technologies).

Proteins whose expression was found down or upregulated by 20% or more *versus* control were then validated by Western blotting analysis.

## Results

### A low grade inflammation is present in Zucker *fa/fa* rat

Zucker *fa/fa* rats display a body mass weight significantly greater than age-matched *fa/+* controls, with a more pronounced development of visceral adipose tissue. These obese animals develop insulin resistance, hyperinsulinemia and moderate hyperglycemia (Table 1).

**Table 1:** Phenotypic features of *fa/fa* and fa/+ rats.

	fa/+	fa/fa
Weight (g)	260.7±11.6	397.4±38.8
Plasma Insulin (ng/ml)	3.66±1.1	60.59±12.9
Plasma Glucose (mM)	7.53±0.11	14.25±1.37

Body weight, plasma insulin and glucose.

To determine whether prediabetic state encompasses an inflammatory process, we identified and quantified a number of circulating pro-inflammatory cytokines and investigated pancreatic islets and ß-cell expression of cytokines and their receptors.

**Figure 1:**
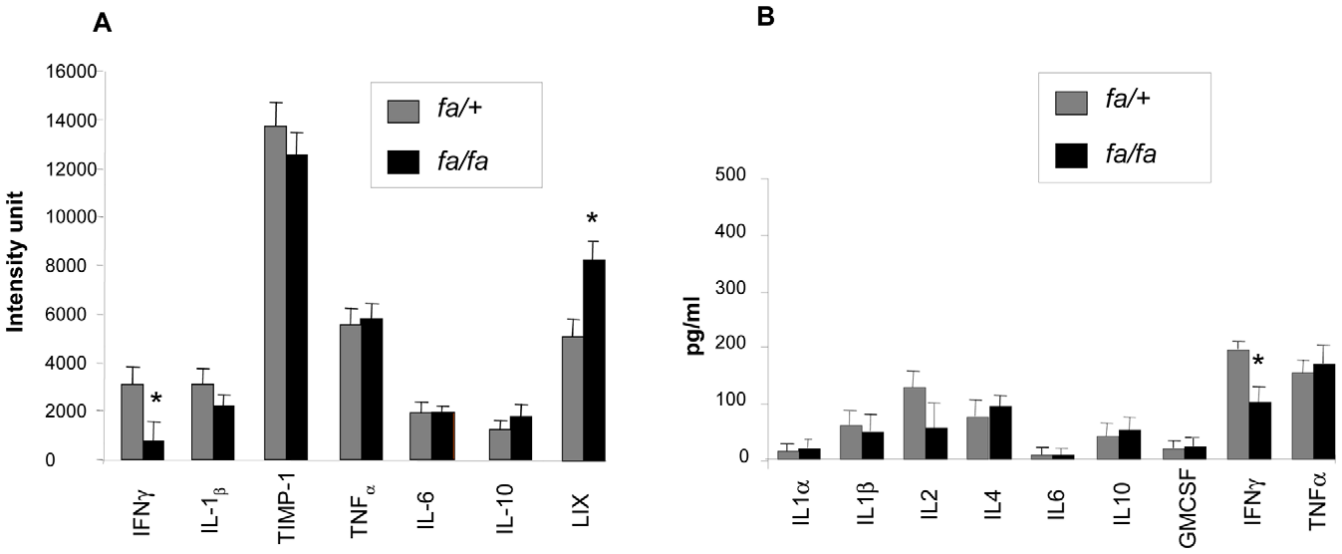
Determination of plasma cytokines levels in *fa/+* and *fa/fa* rats. A) Chemiarray: Rat cytokine array membranes were incubated with 1 ml of 2-fold diluted fa+ or *fa/fa* rats plasma for 2 h. After removing unbound materials, membranes were incubated with a mixture of biotin-labeled antibodies and controls with TBS buffer only. Signals were detected with HRP-conjugated streptavidin and ECL. The relative expression levels of cytokines were determined by densitometry and plotted from 4 *fa/+* and 4 *fa/fa* rats. The array membranes were scanned with a chemiluminescence imager. Signal densities were normalized and background corrected. B) Bio-Plex rat cytokine analysis. Cytokines concentrations were calculated automatically with Bio-Plex Manager software, using a standard curve derived from a recombinant cytokine standard. Concentrations are given in picograms per milliliter.

In blood samples, cytokine expression levels (*fa/fa* versus *fa/+*) were analyzed using the Chemiarray system from Chemicon (Figure 1A) and the Bio-Plex rat cytokine panel from Biorad (figure 1B). Concerning circulating cytokines, most of them were found at similar levels in *fa/fa* and *fa/+* rats; only IFNγ appeared drastically reduced by 50–75% and LIX (CCXCL5) was moderately higher in *fa/fa* rats.

**Figure 2:**
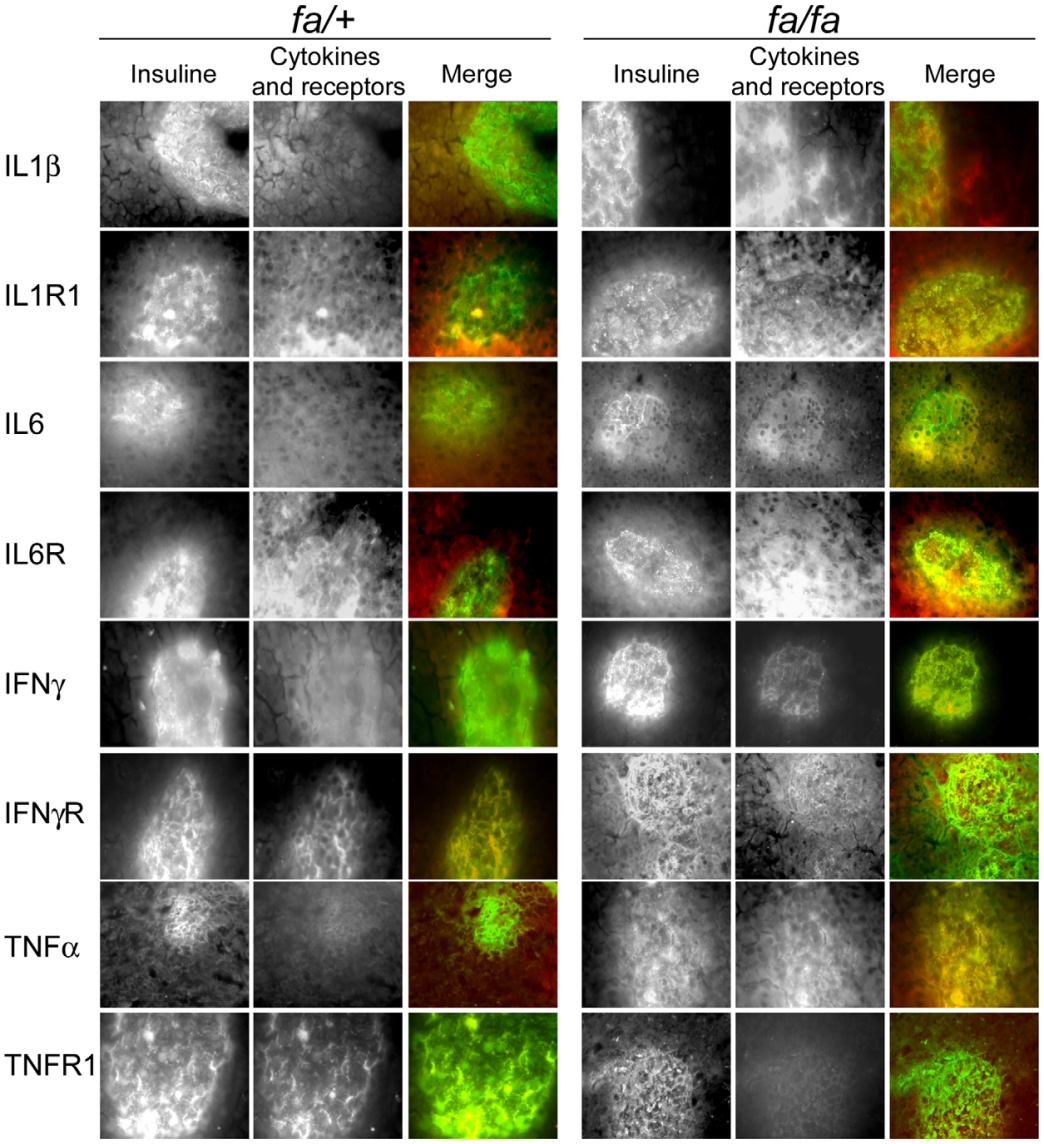
Cytokines and receptors expression on pancreatic sections from *fa/fa* and *fa/+* rats. Pancreatic sections from *fa/fa* and *fa/+* rats were immunostained with anti-insulin and anti-cytokines or cytokine receptors antibodies (anti-TNF-R1, -TNFα, -IL-6R, -IL6, -IL-1R1, -IL-1ß, -IFNγ-R, and –IFNγ). Merge images give the double staining with anti-insulin antibody in green and anti-cytokines or cytokine receptors antibodies in red.

Previous studies point to changes in cytokine production by the liver and adipose tissue in T2D and an increased IL-1ß expression has been recently reported in pancreatic sections of T2 D patients. Therefore, we hypothesized that in prediabetic state, intra-islet expression of inflammatory cytokines could be modified and thus contribute to ß-cell dysfunction in the early phase of T2D. We could confirm this hypothesis by using quantitative RT-PCR and immunofluorescence studies. qPCR experiments were performed on cDNA issued from 12 *fa/+* and 12 *fa/fa* rat islet extracts and repeated three times with reproducible data. Double immunostaining with anti-insulin antibody and anti-cytokine or receptor antibodies was performed on pancreatic tissue and isolated islets to determine expression of cytokines and of their receptors by ß-cells. First, using anti-CD11 antibody, we found no evidence for macrophage infiltration in pancreatic sections (data not shown). IL-1R1 and IL-6R receptors are expressed in both *fa/fa* and *fa/+* rat pancreatic islets but with an overexpression in *fa/fa* endocrine and exocrine pancreatic tissue. Increases in the expression of IL-1ß, TNFα and, to a lesser extent IFNγ, were also observed in *fa/fa* rat islets, attesting that an inflammatory process occurs in pancreas of prediabetic animals (Figure 2).

**Figure 3:**
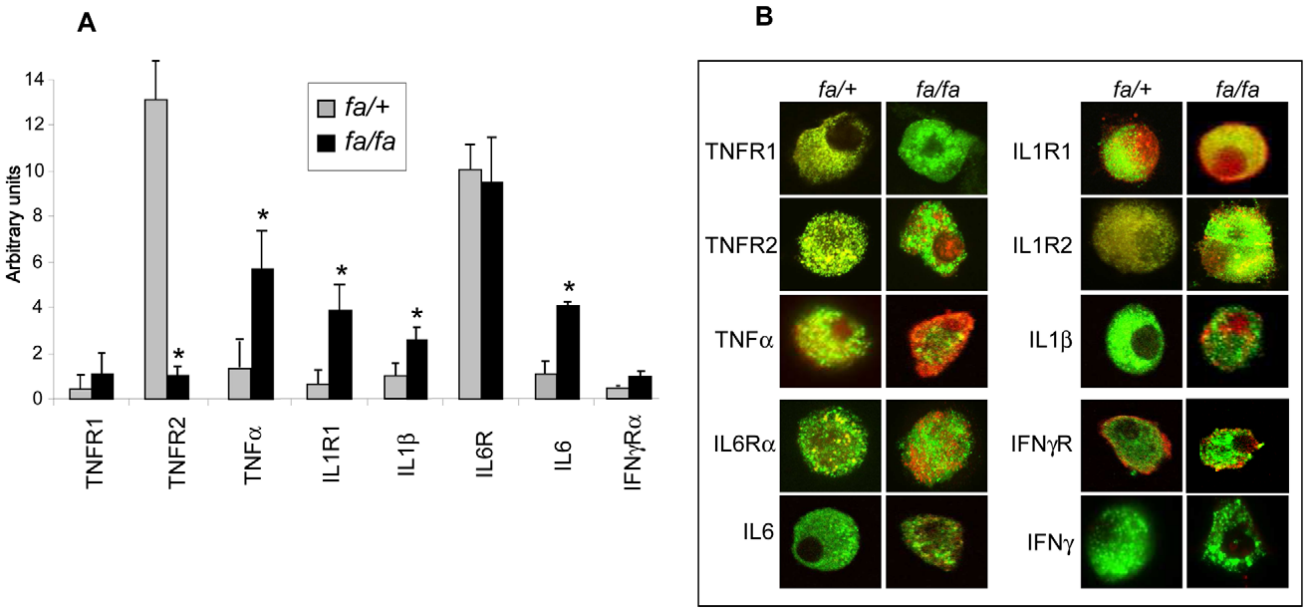
*fa/fa* and *fa/+* rat ß-cells cytokines and receptors expression. A) qPCR analysis of the expression of TNFα, IL-1ß, IL6 and their receptors TNFR1, TNFR2, IL-1R1, IL6R and IFNγRα. Total mRNA were extracted from isolated *fa/+* and *fa/fa* rats islets and converted to cDNA using reverse transcriptase and oligo dT primers. Cytokine or receptor-specific primers were then used to amplify the corresponding cDNA. Each bar represents the average +/− S.E. of twelve *fa/fa* (black square) and twelve *fa/+* extracts (grey square). Asterisk denotes a significant difference between *fa/fa* and *fa/+* extracts. B) Immunocytochemical analysis of cytokines and receptors expression; anti-TNF-R1, -TNF-R2, -TNFα, -IL-6R, -IL6, -IL-1R1, -IL-1R2, -IL-1ß, -IFNγ-Rα, -IFNγ-Rß and –IFNγ antibodies were used in double immunostaining experiments with anti-insulin antibody on *fa/fa* and *fa/+* ß-cells. Staining of cytokines and receptors (red) was detected using Texas red conjugate and insulin (green) was revealed using FITC conjugated antibody. Pictures show merged images of the double staining, from six *fa/fa* and six *fa/+* rat islets.

Interestingly, in *fa/fa* pancreatic islets, IL-1ß and IL-1R1 expressions were found respectively 2.1 and 5.9 fold higher than in *fa/+* rat islets (Figure 3A). Immunostaining of pancreatic ß-cells led to results similar to those observed on pancreatic tissue slices with an increased expression of IL-ß and its receptors IL-1R1 and IL-1R2 on *fa/fa* rat ß-cells (Figure 3B). In addition and very interestingly, we observed alterations in IL-1ß receptor sub-cellular distribution; IL-1-R1 appeared more strongly associated with the β-cell surface and insulin granules in *fa/fa* rats (Figure 3B).

A 2.5 fold higher expression of IL-6 in *fa/fa* versus *fa/+* islets was also observed using qPCR and confirmed by immunofluorescence in pancreatic islets and ß-cells. No difference in IL-6R expression could be detected in *fa/fa* and *fa/+* islets but a clear sub-cellular re-localization of the receptor occurred; indeed most IL-6R appeared co-localized with insulin granules in *fa/+* ß-cells which is no longer the case for *fa/fa* rats.

Finally, TNFα was found over-expressed in *fa/fa* pancreatic islets and immunostaining revealed clearly a cytoplasmic localization of the cytokine in ß-cells. No changes in the expression and staining pattern could be observed for IFNγ; IFNγRβ only faintly expressed in *fa/+* rats was slightly increased and co-localized with insulin in *fa/fa* rats. In summary, qPCR and immunofluorescence studies point to an increase of pro-inflammatory cytokines expression (IL1ß, IL6 and TNFα) in insulin-resistant Zucker *fa/fa* rats with a re-localization of their respective receptors. These results support the hypothesis that pancreatic islets undergo an inflammatory process potentially involved in T2D pathogenesis.

### Signalling pathways involved in the dysfunction of *fa/fa* pancreatic islets

**Figure 4:**
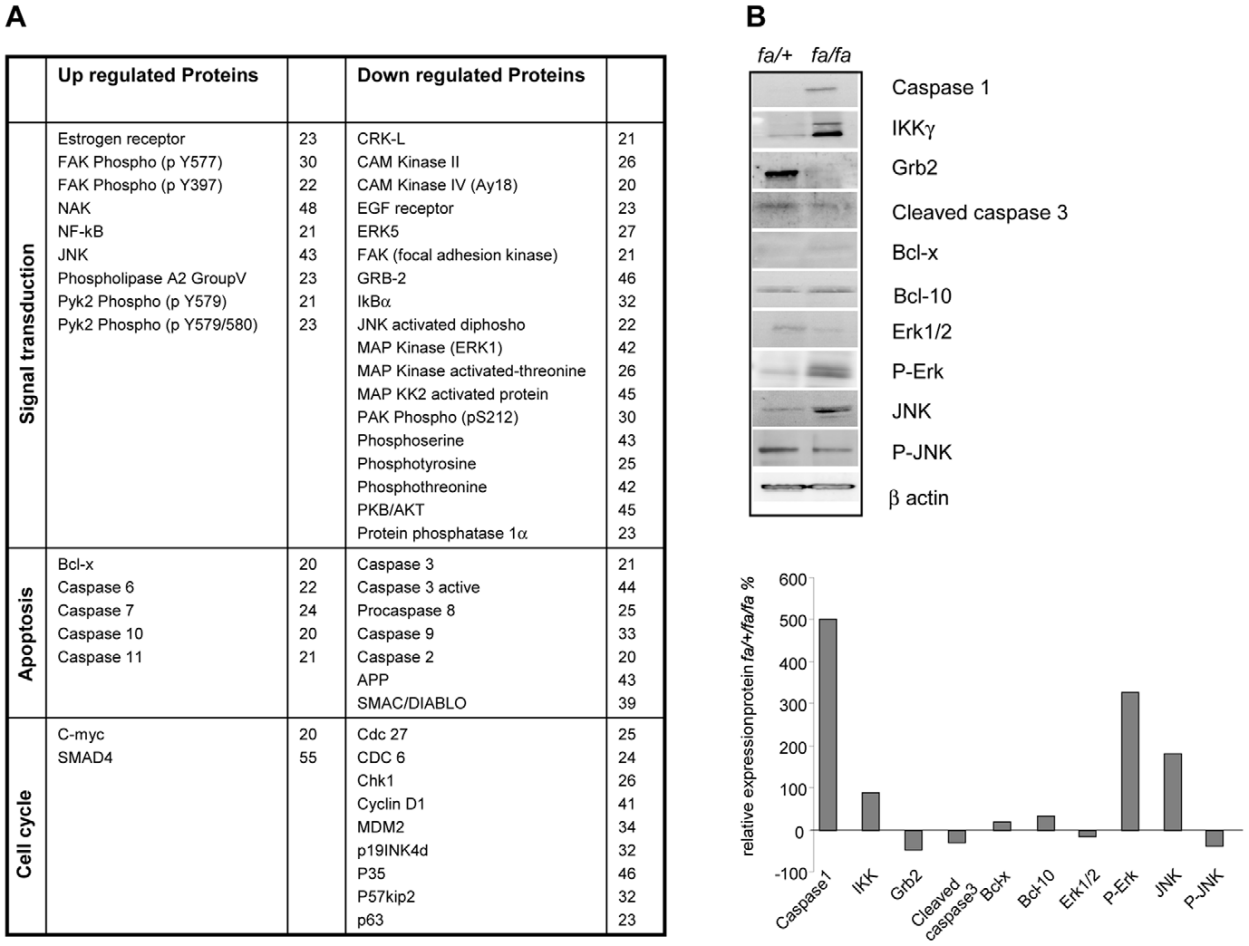
Differential islet proteins expression. A) Protein expression detected by Antibody Array technique. Changes in the expression pattern of proteins involved in signal transduction, apoptosis, and cell cycle were examined by Ab Array. Equal amounts of labelled proteins extracted from *fa/fa* and *fa/+* isolated islets were incubated on nitrocellulose-coated glass slides. Up or down regulated *fa/fa* islet proteins were presented in percentage relative to values recorded for *fa/+* islet proteins. A 20% cut-off value (positive or negative) is considered as significant. B) Immunoblot confirmation of Ab array results. Forty micrograms of total *fa/fa* or *fa/+* islet extracts were run on SDS polyacrylamide gel and blotted. Blots were incubated with Grb2, Caspase 1, Erk1/2, Erk1/2 phosphospecific, JNK, JNK phosphospecific, IKKγ, cleaved Caspase-3, Bcl-10 and Bcl-x antibodies that displayed interesting differential expression in Ab array experiments. Experiments were all duplicated with highly reproducible data. Results were quantified by densitometry as percentage of ß-actin, they are plotted as percents relative to *fa/+* value taken as 0.

We then investigated alterations in the expression of proteins involved in apoptosis, cell cycle, cytoskeleton, nuclear signaling, neurobiology, and signal transduction by Ab array analysis, with more than two hundreds distinct antibodies printed at high-density on a glass microscope slide. We observed differences in protein expression in *fa/fa* versus *fa/+* pancreatic islets for apoptosis, proliferation and NFκB pathways. Among proteins mediating apoptosis [15], caspases, a family of ubiquitous proteases, play a central role. Caspase-3 is situated at a pivotal junction in the apoptotic pathway [16]. It is activated by proteolytic cleavage into 19 and 17 kDa subunits. Our data point to a 44% lower caspase-3 expression in pancreatic islets from *fa/fa* versus *fa/+* rats (Figure 4A). Furthermore, the expression levels of the anti-apoptotic Bcl-x protein and of the pro-apoptotic Bcl-10 protein were found similar in the two phenotypes. Taken together these data suggest that apoptosis is unlikely to be involved in inflammation-induced ß-cell dysfunction in prediabetic state. In contrast, we observed functionally relevant differences in the expression of factors involved in pancreatic islets cell proliferation; indeed, a 46% lower expression of Grb2 which plays a major function in proliferation of various cell types, could be recorded in islets from *fa/fa* rats. Interestingly, Erk1, and MAPK phophorylated form on threonine appeared also down-regulated by respectively 42%, and 26% in *fa/fa* versus *fa/+* pancreatic islets. In addition, JNK was found up-regulated, and phospho-JNK decreased by 22% in *fa/fa* rats. To better assess the extent of the differences in GrB2, Erk and JNK expression, we performed a Western blotting analysis for these factors (Figure 4B). Comparison of the bands confirms that Grb2, and phospho-JNK expressions are significantly depressed in pancreatic islets of *fa/fa* rats when compared to controls, whereas phopho-Erk is up-regulated. Cleaved caspase 3 was down-regulated and anti and pro-apoptotic proteins Bcl-x and Bcl-10 remained unchanched, in agreement with the Ab array results. Taken together, these data suggest an alteration of islets cell proliferation in *fa/fa* rats without major effect on cell viability. Furthermore, modifications in the expression of proteins involved in cell cycle were also observed with a decrease of cyclin D1 and regulating proteins as well as an increase in SMAD4 and a decrease of amyloid precursor protein (APP) of 43%.

Of great interest are also modifications in the expression of proteins involved in IL-1β signaling pathways which were particularly affected in *fa/fa* rat islets; indeed, NAK, Iκκ kinase, NFκB and c-Jun/AP-1 were found increased by respectively 45, 20 and 30% with a decrease of IκBα in *fa/fa* versus *fa/+* islets (Figure 4A and B).

### Effects of IL-1ß on insulin release and apoptosis from the isolated *fa/fa* and *fa/+* rat islet

**Figure 5:**
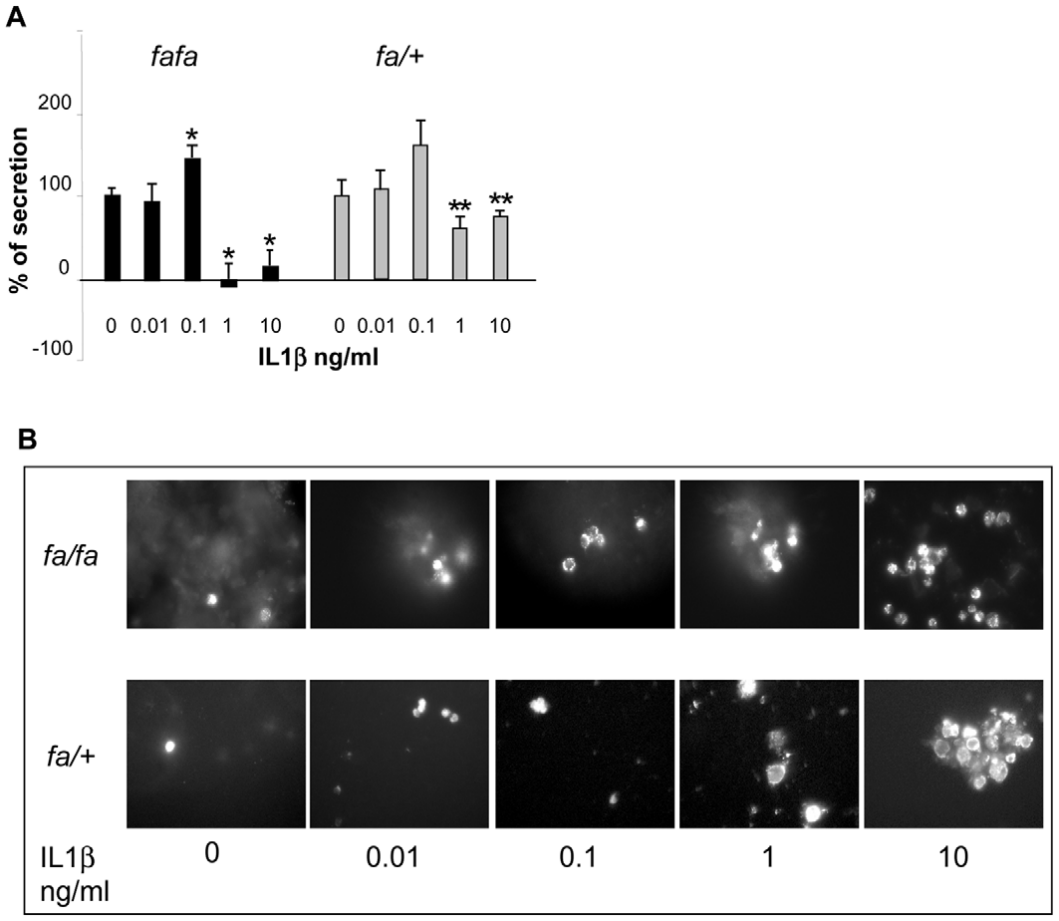
Effects of IL-1ß on the functionality of *fa/fa* and *fa/+* rat islets. Rat islets were cultured for 2 days at 7.5 mM glucose for *fa/fa* rat islets and 10 mM glucose for *fa/+* rat islets in the absence and in the presence of increasing IL-1ß concentrations. Rat islets functional activity was then determined by insulin release and apoptosis measurements. A) Changes in the differential insulin release at 2.8 and 8.3 mmol/l glucose, by *fa/+* and *fa/fa* isolated islets rats previously exposed during 48 h to increasing IL-1β concentrations. Control value (given as 100%) represents the increase in insulin secretion observed in islets cultured in the absence of interleukin. *fa/fa* *: P<0.05 versus untreated islets, *fa/+* **: P<0.05 versus *fa/fa* rat islets. Data were collected from three separate experiments, each performed with four *fa/fa* and four *fa/+* rats). B) Islet β-cells apoptosis determined by annexin immunofluorescence staining.

The increased IL-1ß expression in ß-cells in *fa/fa* zucker rats prompted us to study the effect of an exposure to IL-1ß of *fa/fa* and *fa/+* rat islets on insulin release and β-cells apoptosis. *fa/fa* and *fa/+* rat islets were cultured in the presence of increasing IL-1ß concentrations for 2 days (Figure 5A). Previous exposure to 0.01 ng/ml did not modify the difference in insulin secretion between islets incubated at 2.8 and 8.3 mmol/l glucose. After 48 h culture with 0.1 mmol/l cytokine, the differential insulin release was increased by 35 and 49% in respectively *fa/fa* and *fa/+* islets. In contrast, exposure to higher concentrations, 1 and 10 ng/ml, resulted into an almost complete (80%) inhibition in *fa/fa* islets versus only 33 and 23% in *fa/+* islets (P<0.05). Insulin content was not significantly affected (data not shown). We also measured the effect of IL-1β on islet-cell viability using confocal microscopy after annexin staining. After the 2-day culture in the presence of increasing concentrations of IL-1ß, only moderate differences in the number of apoptotic cells could be observed in *fa/fa* versus *fa/+* islets (Figure 5B). Likewise, flow cytometry analysis pointed to a small 17% increase of dead ß-cells in *fa/fa* versus *fa/+* islets (data not shown).

## Discussion

Recently cytokines have been recognized as essential factors involved in nutrient-induced β-cell dysfunction during the development of type 2 diabetes; however that pancreatic islets are the target of inflammation and undergo a local inflammatory process in prediabetic states remains poorly explored. In the present study, if only minor changes in systemic markers of inflammation could argue for the presence of a low grade inflammation state, we bring evidence for the development of a clear inflammatory process in pancreatic islets from Zucker *fa/fa* rats, a model of obesity-associated insulin resistance.

Concerning circulating inflammation markers, we unexpectedly found a decrease in IFNγlevels in our obese insulin resistant rats. As this cytokine attenuates insulin signalling in adipose tissue [17], the decrease in IFNγ could participate in and favour the development of fat stores in these animals. Conversely, we also observed an increase in circulating neutrophil chemoattractant LIX (CXCL5), which is known to induce IL1-β and TNFα promoter activity, cytokine gene expression and to activate NFκB [18]. This chemokine could thereby amplify the inflammatory cascade and participate in the local production of cytokines.

Our qPCR and immunocytochemical studies on pancreatic islet β-cells bring evidence for a marked induction of IL-1 production together with a pronounced increase in IL1-R1 expression in *fa/fa* rats. Our data are in line with those of previous studies pointing to an increase in IL-1β expression in islets from T2D patients [12], hyperglycemic Psammomys obesus gerbils as well as OLEFT [19] and GK [20] diabetic rats. Hence, we extend the latter observations to the prediabetic state like in the obese insulin resistant *fa/fa* rat. Interestingly, induction of IL-1β has also been reported to occur in human islets (from non diabetic organ donors) upon culture in high glucose and to be followed by NFκB activation. In our study, we observed an increased expression of NFκB activating kinase (NAK), known to induce IKB degradation, to increase NFκB activity, and thus to feed an auto-stimulatory loop of IL-1β expression through activation of the cytokine promoter as previously suggested in T2D patients [13]. In this respect, our data show that, in addition to the increased IL-1R1 expression, the receptor also appears re-localized and more associated with the β-cell plasma membrane which could also contribute to a possible autostimulation of IL-1β. This effect might also be further sustained by our findings of first an increased expression of caspase-1, required to cleave pro-IL-1β to active IL-1β and second decreased levels of APP the precursor molecule of amyloid, also known to potentiate IL-1β processing.

As concerns β-cell function, IL-1β is known to induce a bimodal effect on insulin secretion: a stimulating and a suppressive effect depending on IL-1β concentration, duration of exposure and glucose concentration [21], [22]. The secondary inhibitory phase is known for a long time [23], [24] and is accompanied by decreases in oxidative metabolism and calcium uptake [25] secondary to nitric oxide (NO) production after induction of the inducible form of NO synthase (iNOS) [26], [27]. At the opposite, the initial stimulatory effect of IL-1β has been shown to be glucose-dependent and related to diacylglycerol formation and stimulation of PKC [28]. As for β-cell survival, IL-1β induces apoptosis in rodent and human islets [24], [29], [30] but the cytokine has been reported to stimulate β-cell proliferation and to inhibit apoptosis at low concentrations [31]. In our study, we confirm the bimodal effect of IL-1-β on insulin secretion in both *fa/fa* and lean control Zucker rats. Interestingly, islets from obese insulin resistant rats seem to be more responsive to both stimulating and inhibitory concentrations of the cytokine. Such a difference could probably be related to the increased expression and plasma membrane localization of IL1-R1 and be of physiological relevance in IL-1β autocrine regulation of β-cell function. In this respect, we cannot exclude that the stimulating effect of IL-1β on β-cell function could play a part in the high plasma insulin levels that compensate for insulin resistance in obese rats. Increased IL-1β and IL1-R1 expressions have no impact on β-cell survival under basal conditions in *fa/fa* rats. Furthermore, we found no significant difference in the apoptotic effect of the cytokine in *fa/fa* versus lean controls.

TNFα has been proposed to be a key compound of the obesity-diabetes link [32]. The cytokine is over-expressed in adipose tissue of different models of obesity and known to inhibit insulin signalling. Moreover, immuno-neutralization of TNFα in Zucker *fa/fa* rats has been shown to increase insulin receptor auto-phosphorylation and phosphorylation of insulin receptor substrate-1 (IRS-1) in muscle and adipose tissue and to reduce glucose, insulin and FFA plasma levels. In our study, we now demonstrate, for the first time, a very strong increase in TNFα expression in pancreatic β-cells from *fa/fa* rats. That such an increase could interfere in β-cell function cannot be excluded; its importance should nevertheless be dampened by the drastic decrease in TNF-R2 receptor expression and its delocalization; the receptor seems much less co-localized with insulin granules. The increased expression of TNFα could however be partly responsible for the marked increase in IL-6 expression we found in pancreatic β-cells; indeed, TNFα has been reported to up-regulate IL-6 in murine pancreatic islets [33]. No consistent in vitro data are available regarding insulin secretion in human and rodent islets [34]. However, the marked increase in IL-6 expression together with a clear delocalization to insulin granules questions the possible involvement of IL-6 in the hyperinsulinemia of *fa/fa* rats, which deserves to be reassessed in vivo in this model of prediabetic state. Concerning β-cell survival, IL-6 has been shown to stimulate human islet cell proliferation [35] and to afford protection against IL-1β, TNFα and IFNγ-induced cell death [36]. Such an effect could occur in pancreatic islets and account for the marked decrease in active caspase-3 expression; indeed, chronic exposure of neurons to IL-6 prevents the enhancement of the cleaved caspase-3 levels induced by NMDA [37].

Finally, from our abArray study, it appears that up- and down regulation of factors involved in the regulation of cell proliferation/survival, contributes to control islet hyperplasia known to occur in *fa/fa* rats [38].

We may conclude that pancreatic islets from hyperphagic, obese insulin-resistant Zucker *fa/fa* rats undergo a clear and possibly self-perpetuating inflammatory process. The complexity of cytokines effects and of their interactions makes it difficult to evaluate their pathogenic role in β-cell hyperactivity that compensates for insulin resistance. In Zucker rats, compensation will keep going, but in the presence of an additional β-cell defect, as in ZDF rats, inflammation will be exacerbated and diabetes will ensue.
